# Antibacterial Efficacy and Biocompatibility of Denim Fabrics Finished with Plant-Based Nanoemulsions Using Mechanical Finishing and Digital Printing

**DOI:** 10.1021/acsomega.5c07941

**Published:** 2026-01-09

**Authors:** Prabhuraj D. Venkatraman, Usha Sayed, Swati Korgaonkar, Sneha Parte, Holly Ansell-Downey, Jonathan A. Butler, Tuser T. Biswas

**Affiliations:** † Manchester Fashion Institute, Faculty of Arts and Humanities, 5289 Manchester Metropolitan University, Cavendish Street, Manchester M15 6BG, U.K.; ‡ Department of Fibers and Textile Chemistry, 80493 Institute of Chemical Technology [ICT], Nathalal Parekh Marg, Matunga, Mumbai 400 019, India; § Department of Life Sciences, Faculty of Science and Engineering, Manchester Metropolitan University, Chester Street, Manchester M1 5GD, U.K.; ∥ Department of Textile Technology, Faculty of Textiles, Engineering and Business, 1802 University of Borås, Borås 501 90, Sweden

## Abstract

This research examines mechanical finishing and digital printing methods for imparting antibacterial properties to denim fabrics. It evaluates the use of plant-based nanoemulsions, which are nontoxic and environmentally friendly, as alternatives to synthetic antimicrobial agents. This finishing technique enhances the functional properties of denim fabrics, enabling them to be used for longer periods without requiring frequent washing. Additionally, it prevents the formation of odor and microbial growth during consumer use. Two types of nanoemulsions, namely, Karanja and Shankapushpi, were derived from plant-based herbs combined with coconut oil and curry leaves. The nanoemulsions were characterized for their thermal stability, particle size, and percentage add-on. The finished denim fabrics were assessed for their antimicrobial properties using Gram-positive bacteria (*Staphylococcus aureus*) and Gram-negative bacteria (*Escherichia coli*). Furthermore, the durability and skin safety of the finished fabrics were tested. The antimicrobial efficacy of Karanja nanoemulsion before washing was 99.73% (*S. aureus*) and 99.74% (*E. coli*), and for Shankapushpi, it was 99.77% (*S. aureus*) and 99.73% (*E. coli*). For digitally printed denim, no increase in bacterial growth was observed after 24 h. After washing, only a marginal reduction in the antibacterial efficacy (>99.2%) of the finished denim fabrics was observed, demonstrating the durability of the finish. In vitro cytotoxicity assessments demonstrated a cell viability of >70%, indicating acceptable cytotoxicity of the denim fabric and safety on the skin. Fourier transform infrared spectroscopy (FT-IR) analysis revealed the presence of a triple-bond carbon at 2105 cm^–1^ and fatty acids at 3006 cm^–1^ in both the nanoemulsions, Karanja and Shankapushpi, which are responsible for the antimicrobial property. This research suggests that denim fabrics can be treated with durable antibacterial properties using sustainable, environmentally friendly, and biocompatible plant-based herbal nanoemulsions. The digital printing method that uses fewer resources demonstrated high precision in applying the nanoemulsion to the fabric and proved more efficient than mechanical methods. This research introduces innovative approaches to enhance denim fabrics by preventing unpleasant odors from microbial growth, disinfecting surfaces, and reducing the frequency of washing. These methodologies employ plant-based herbal treatments for the first time to enhance denim functionality, highlighting potential applications in sportswear and athleisure that prioritize freshness, durability, and sustainability.

## Graphical abstract

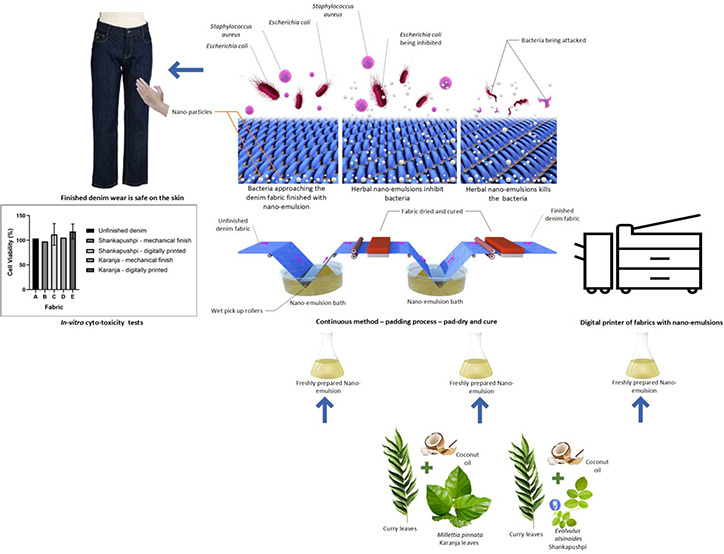

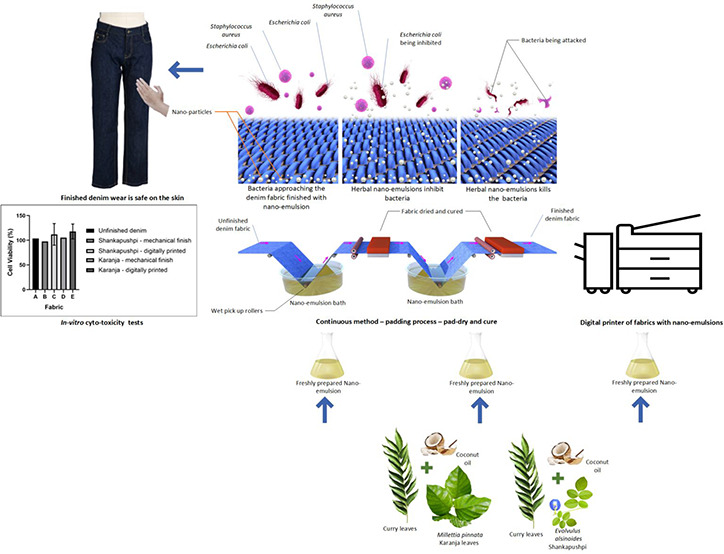

## Introduction

Functional finishing of textiles with antimicrobial properties has been a focus for industry and academic professionals over the past decade, particularly in the postpandemic era. The trend toward increased sanitation and improved lifestyle has also led to a rise in demand for antimicrobial finishing more recently. Researchers have shown progress in surface finishing textiles using various synthetic organic compounds. Due to the recent global pandemic and rise in awareness of improving health and preventing infections, antimicrobial finishes on fabrics are experiencing phenomenal growth. The functional finishing of materials is currently facing an upward market trend. The antimicrobial textiles market is expected to reach $12.3 billion by 2024 at a compound annual growth rate (CAGR) of 5.4% between 2019 and 2024.^1^ Among many functional finishes, durable/wrinkle resistant, temperature regulation, flame retardant, repellent/release, and the demand for biological control finishes such as antimicrobial or anti-inflammatory are increasing.^2^ There is a continuous demand for antimicrobial finishes. Recently, Amicor fibers—a cobranding between Thai Acrylic Fiber (Birlacril) and Sanitized AG—reported antibacterial and antifungal additives added during fiber production, which is aimed to benefit asthma and allergy sufferers and is suitable for innerwear, sportswear, and socks.^3^

Textiles made from cotton fibers retain moisture, offer a larger surface area, and absorb moisture from the skin and temperature from the body.^4^ These conditions are ideal for the growth of bacteria and fungi, resulting in odor and potential spreading of infection through clothing. Infections spreading through workwear garments^5^ and linen^6^ were noted as being significant risk factors. Bacterial species can survive on cotton-based textiles at room temperature for extended periods, for instance, *Staphylococcus aureus* (up to 8 weeks) and *Escherichia coli* (up to 45 days in mixed fibers).^7^ In addition, higher air humidity enhances the survival of *E. coli*, and low air humidity improves the survival of *S. aureus*.^7^ Functional finishes incorporating antimicrobial agents have become mandatory to prevent the spread of infections through textiles, particularly in applications such as medical and hygiene products, sportswear, and casual wear. Among all other textile products, denim fabric is widely used by people of all ages due to its durability and comfort. It is extremely popular for leisure and, more recently, semiformal and casual wear.

Functional finishes improve the appearance, fabric handle, care and maintenance, comfort, and apparel protection.^8^ These finishes enhance the fabric properties and impart value to the product using various techniques, such as mechanical, chemical, and biotechnological methods. Several functional textile finishes have been reported.^8,9^ Some specific techniques to develop functional finishes on fabrics include the immobilization of enzymes, nanocoatings, the use of plasma, and layer-by-layer deposition.^9,10^ More recently, encapsulation techniques to impart bactericidal and insecticidal properties were reported. Microencapsulation involves a polymeric film or shell, which captures the liquid or solid substances, called the core material.^11^ Other forms of microencapsulation—phase separation, suspension cross-linking, and complex coacervation—were also reported.^12^ Foam finishing and blade-on-air coating systems were also reported.^13^ The core materials can be essential oils, proteins, phenolic compounds, and aromatic compounds. Studies suggest that essential oils and aromatic compounds exhibit antibacterial, antiviral, and antifungal properties.^14,15^ A more comprehensive analysis of the fabrication of functional textiles was also presented earlier.^16^ There has been an increasing trend toward denim wear that washes less and stays fresher, especially when finished with compounds that resist viruses and microbes. The postpandemic market has driven the growth of new technologies, such as HeiQ’s Viroblock NPJ03, an antiviral technology added to laundry to sanitize and create a germ-resistant effect.

Similarly, Diesel partnered with Polygiene, which launched its 2021 denim collection “ViralOff” to resist the COVID-19 virus.^17^ Turkish denim mill Calik Denim also reported an antimicrobial denim collection for Fall/Winter 2021 using Washpro technology.^18^ The market has been steady, as denim has been a staple fashion piece. However, the market has experienced a downward trend since 2017, driven by rising athleisure demand and the COVID-19 lockdown, which led consumers to opt for more comfortable loungewear over denim. However, the denim wear market is predicted to grow at a CAGR of 1.9% between 2019 and 2024, especially as life becomes more social and active and as innovations in denim finishing^17^ continue.

Several antimicrobial agents for textile applications, such as silver, iodine, and natural organic antimicrobial agents—chitosan, aloe vera, manuka honey, essential oils, and bark cloth—were reported.^19^ Various mechanisms of finishing textiles with antimicrobial properties and release mechanisms were also discussed.^20^ Synthetic agents such as quaternary ammonium compounds (QAC), silver nanoparticles, triclosan, metal and metallic salts, PHMB (poly hexamethylene biguanide), and *N*-Halamines^21^ are used for finishing textiles with an antimicrobial finish. Quaternary ammonium compounds [QACs] are widely used for antimicrobials, surfactants, and preservatives, including textiles, which resist a range of Gram-positive and Gram-negative bacteria.^22,23^ They are used in finishing cotton, polyester, nylon, and wool-based textiles.^21^ QACs are also used in cleaning, sanitizers, and personal care products (wipes); exposure to these agents is possible. There have been reports of dermal effects of QACs, such as skin irritation, sensitization, dermatitis in contact with personal care products, and inhalation of contaminated dust.^24^ It has been reported to be toxic to aquatic organisms (fish, daphnids) found through wastewater systems.^25^ Metal-based antimicrobial finishing (silver, copper, zinc, and cobalt) is environmentally toxic.^26^ Similarly, triclosan, used as an antimicrobial agent in textiles to prevent foul odor and microbial growth, is also bound to affect human health (affecting eyes, respiratory system, and skin) and the environment.^26,27^ Triclosan can be absorbed through the skin, nose, and mouth when humans come into contact with products finished with the agent. Triclosan affects hormones in the body. For instance, it can affect androgen in men and estrogen in women.^28^ It also reportedly triggers breast cancer in women.^26^ Hence, a pressing rationale exists to identify and develop antimicrobial agents that have the least negligible impact on human health and the environment. Natural compounds derived from plants and herbs [leaves, roots, fruits, seeds, bark] are nontoxic, widely available, and environmentally friendly.^29,30^

Previous studies have highlighted the potential of various plant-based herbal combinations, such as basil (*Ocimum Tenuiflorum Linn*—Holey Basil, or commonly called Tulsi).^31,32^ *Viola odorata* flower and *Tinospora cordifolia* stem have also shown good antibacterial and antifungal activity and are used to treat various diseases due to the presence of flavonoids, phenols, alkaloids, and saponin compounds.^33^ Multiple combinations of plant extracts from *Aegle marmelos*, *Plumbago zeylanica*, and *Rhinacanthus nasutus* were tested for their antimicrobial properties against pathogens.^34^ Phytochemical or bioactive compound screening identified alkaloids, flavonoids, tannins, saponins, and terpenoids in leaf extracts of eucalyptus and lemongrass.^35^ These compounds offer excellent antibacterial properties. Similarly, aqueous neem extract (*Azadirachta indica* A. Juss) also showed excellent antibacterial activity against Gram-positive bacteria—*S. aureus*—due to active limonoids.^36^ Several compounds were also identified in curcumin, aloe vera, onion (Allium cepa), clove oil, and eucalyptus oil.^37,38^ Interestingly, carvacrol essential oil and carboxybetaine zwitterionic moieties were also combined in the development of biobased polyurethane with antibacterial properties.^38^ The use of natural dyes obtained from walnut shells, onion peels, tansy, and Hypericum wildflowers was finished onto polycotton fabrics with and without mordant, showing antibacterial and antifungal activity.^39^ More recently, a combination of tea polyphenols and phytic acids has also been reported on viscose fabrics for bacteriostatic properties.^40^ Natural dyes containing polyphenols, such as pyrogallol, phloroglucinol, and pyrocatechol, have been used to dye textiles, including cotton and wool, with antibacterial properties.^41^

Denim fabrics (100% cotton) have previously been microencapsulated and nanoencapsulated with herbal extracts (*Ricinus communis, Senna auriculata, Euphoria hirta*) using the spray method with bovine albumin fraction as a wall and nanoparticles as a core material and a coacervation process, respectively. The finished fabric exhibited antimicrobial activity against *S. aureus* and *E. coli* after 20 washes.^42^ Previous research on finishing organic cotton fabrics (plain weave) using continuous and exhaust methods, using a combination of curry leaf, coconut oil, *Moringa oleifera*, and *A. marmelos*, showed excellent antimicrobial activity.^43^ The antibacterial efficacy against microorganisms was in the range of 99.24–99.78% before washing and 99.26–99.03% after 20 washes. The nanoemulsions were produced using plant-based herbs *Millettia pinnata*, coconut oil, and curry leaves and *Pedalium murex*, coconut oil, and curry leaves. Antimicrobial resistance was in the range of 98.62–99.87%, even after ten washes, indicating that the finishes were effective and durable.^44^ *Evolvulus alsinoides*, commonly called “Shankapushpi”, is an ayurvedic herb that possesses antioxidants, which have shown many health benefits, including treating bowel problems and improving memory and mental function.^45,46^ GC–MS analysis revealed the presence of phytocompounds tetradecanoic acid and *n*-hexadecanoic acid, which possess antimicrobial properties.^47^ The above data show that plant-based herbs contain bioactive compounds. However, these herbal extracts have not shown potential for denim fabrics or their durability. Herbal extracts have been incorporated into textile surfaces using padding-based finishing methods, which require a substantial volume of extract solution. Some of this solution must be drained after the padding process. Digital printing can reduce such waste, as it is a direct solution application method and requires only a few milliliters of extract solution compared to the conventional padding process.^48^ Digital printing can also save incubation time, drying time, and electrical energy compared to padding processes. Additionally, by using digital printing, it is possible to add herbal extracts to a specific part of a garment instead of applying them to the entire area and create necessary design or functional patterns.^49^

Therefore, the primary purpose of this current research was to examine the finishing of denim fabrics using a continuous method (pad-dry-cure) and digital valve-jet printing with a combination of nanoemulsions derived from *Millettia pinnata*, coconut oil, and curry leaves (nanoemulsion 1) and *Evolvulus alsinoides*, coconut oil, and curry leaves (nanoemulsion 2). In addition, various parameters, including percentage add-on, thermal stability, pH, antibacterial efficacy (before and after ten washes), surface morphology, color analysis, energy-dispersive X-ray spectroscopic analysis (EDX), attenuated total reflectance–Fourier transform infrared spectroscopy (ATR-FT-IR), X-ray diffraction analysis, in vitro cytotoxicity, and tensile strength, have been explored. The finished denim fabric was also developed into a garment and evaluated for its fabric drape and garment fit on the mannequin.

## Methods and Materials

Based on an extensive review of resources and previous research,^43,44^ this study used a combination of plant-based herbs, curry leaf, pure, odorless coconut oil, and nonionic surfactant. In this study, a heavyweight denim fabric was chosen for durable antimicrobial finishing with nanoemulsions, as denim is considered a staple product among many, and a functional antibacterial finish can enhance its value. The fabric was a 3/1 twill-weave fabric structure (warp-faced). The fabric has a distinct face side with indigo-dyed (dark blue) warp yarn interlacing with the white-colored weft yarns (Figure 1). The physical details of the fabric are given in Table 1. The fabric was sourced from Sanjay Shah Associates, Mumbai, India. The leaf herbs used in the study were *Millettia pinnata* (Karanja) and *Evolvulus alsinoides* Linn. (Shankapushpi). The FDA-approved surfactant, polysorbate monobate 80, was sourced from Loba Chemicals, Mumbai, India, while ethanol was sourced from Himedia Chemicals, Mumbai.

**Figure 1:**
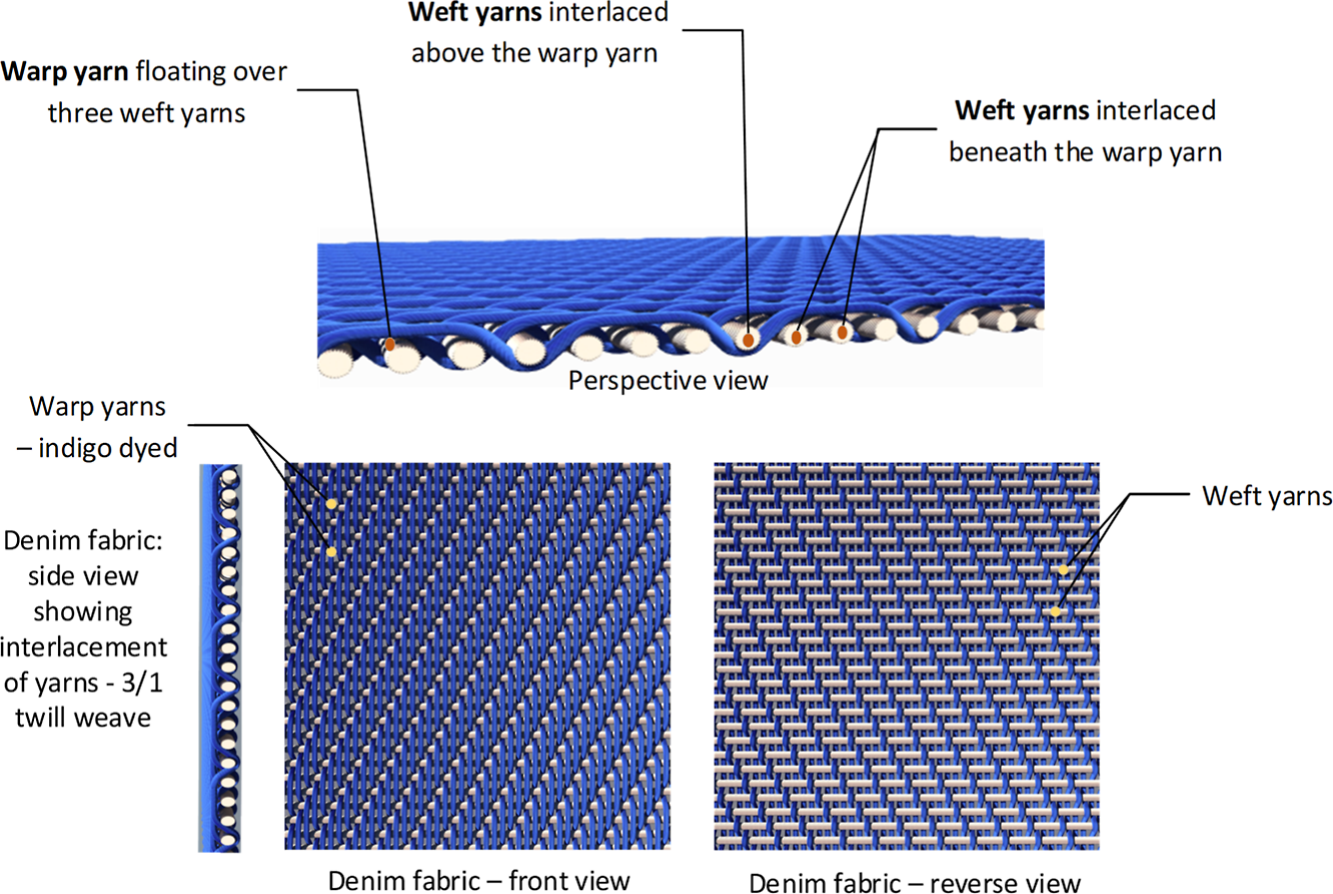
Visual illustration of denim fabric—3/1 twill weave.

**Table 1:** Physical Properties of the Denim Fabric

parameter	values	notes
fabric structure	3/1	twill weave—warp faced; three warp yarns float over weft yarns, giving a diagonal line appearance on the fabric
fabric weight (g/m^2^)	409.27 (±5.29)^a^	measured by weighing fabric (10 × 10 cm) specimens on a balance
fabric thickness (mm)	0.73 (±0.03)^a^	fabric thickness—the distance between two plates
bulk density (g/cm^3^)	0.56	it is the weight per unit volume of the fabric
fabric count (EPI X PPI)	76 × 61	ends per inch (no. of warp yarns) × picks per inch (no. of weft yarns)
cover factor *K* = *k*_1_ (warp) + *k*_2_ (weft)	26.83 + 20.22	it is the area of a fabric covered by a set of threads
yarn count warp/weft (tex)	80/71	yarn count denotes the linear density; it is determined by weighing (g) a specified length of yarn (1 km) – tex

a Number in the parentheses indicates standard deviation.

### Herbal Extraction and Steam Distillation

The herbs were washed thoroughly with distilled water and dried at 105 °C to remove all of the dirt and impurities present. Two different types of oil mixtures were extracted through the steam-distillation method. These herbal mixture combinations include (1) *Evolvulus alsinoides*, curry leaf, and coconut oil and (2) *Millettia pinnata*, curry leaf, and coconut oil. A 10 g amount of each herb was dried in sunlight. To this dried herb powder, 5 g of curry leaves and 100 mL of coconut oil were added, and the mixture was boiled. Plant material was heated using steam from a steam generator. During this process, the amount of heat applied plays a vital role in determining how effectively the plant material structures break down, burst, and release aromatic components, or essential oils. This technique increases isolated essential oil yields and reduces wastewater production during the extraction process. All oil mixtures were extracted in the same way. The prepared mixture was then further processed for oil extraction using a Soxhlet extractor with ethanol as the solvent. When the solvent evaporated, the oil was filtered and collected. The extracted oil was stored in a glass bottle until further analysis.

### Preparation of Nanoemulsions

Nanoemulsions were prepared using the above herbal oil mixtures. The herbal oil emulsions were prepared in a 1:1 ratio for all four oil mixtures. Previous research highlighted that a 1:1 ratio was appropriate and better than other ratios, 1:0.5, 1:5, and 1:2, where oil to surfactant was in equal proportions.^33^ In the 1:1 ratio, 100 mL of distilled water, 1 mL of oil mixture [three parts of *Evolvulus alsinoides*, two parts of curry leaves, and one part of coconut oil], and 1 mL of polysorbate were used. Nanoemulsions of these mixtures were prepared using a high-speed homogenizer (Manufacturer Tool-Tech) at 1000 to 5000 rpm. The homogenization was performed for 1 h for each herbal oil mixture to obtain a stable emulsion. Furthermore, optimization and characterization studies have been conducted on these emulsions.

### Mechanical Finishing of Fabric with Nanoemulsions

The denim fabric was finished using the prepared nanoemulsions via continuous processing methods, such as the pad–dry–cure method. A predetermined denim fabric sample has been finished for further analysis. The fabric had been padded using the two-dip and two-nip method at 75% expression [the rate at which the fabric is passed through the padding mangle]. The padded fabric was then dried at 80 °C and cured at 140 °C. Padding had been carried out for the herbal oil emulsion mixtures. The pH of the nanoemulsions was determined using a standard pH meter (EquipTronic) at 37 °C. The particle size of the nanoemulsions was also determined using a particle size analyzer (Shimadzu SALD-7500 nano, Kyoto, Japan). The thermal stability of the nanoemulsions was determined using a Metal-Lab MSI-17B (Metal-Lab Scientific Industries, Mumbai, India) at varying temperatures, observing the oil separation from the constituents.

### Digital Printing

Two separate inks were made by mixing 70% Karanja and Shankapushpi emulsions with 30% glycerol as a viscosity modifier. A digital valve-jet printer (Chromojet tabletop CHT-TT-110, J. Zimmer Maschinenbau GmbH, Austria) was used in this work. A printhead with eight nozzles, each having a diameter of 120 μm, was used for printing. Inks were supplied directly to the printhead from stainless steel bottles via inert plastic tubing. The ink was printed onto fabric using a switchable electromagnetic valve in the printhead, combined with pressurized air. The printhead can move in three dimensions over a stationary support to place the fabric. It can print over an area of 30 × 30 cm on fabrics up to 5 cm thick. The printing speed, air pressure, and image resolution were maintained at 1 m s^–1^, 0.5 bar, and 76 dots per inch, respectively. An image of a solid block was printed on all of the samples. A few samples were printed for several passes, as detailed in the result section. During a single pass on a 30 cm × 30 cm area, about 4–5 mL of ink was printed on the fabric samples. After ink preparation and printer setup, it took about 5 min to print one sample and consumed about 0.021 kWh of energy. Samples were then dried at room temperature for 1 h.

### Fabric Characterization

The surface morphology of finished denim fabrics was determined using scanning electron microscopy (SEM, Carl Zeiss Supra 40 VP, Oberkochen, Germany); a variable pressure of 30 Pa was used. In addition, energy-dispersive X-ray spectroscopic analysis (EDX) was carried out using an Apollo 40 SDD (Tilburg, the Netherlands). The elemental composition of finished denim fabric with nanoemulsions has been identified using EDX analysis. In addition, fabric samples were characterized using Agilent Technologies, US. Fourier transform infrared spectroscopy (FT-IR) interfaced with an attenuated total reflectance (ATR) sampling accessory with a single-bounce diamond crystal. The FT-IR spectra for denim fabrics were recorded from 4000 cm^–1^ to 600 cm^–1^ by accumulation of 64 scans at a spectral resolution of 4 cm^–1^.

### Fabric Color Analysis

Color characteristics were analyzed using a Datacolor Spectrophotometer [DC 700]. The following parameters were examined: CIE Lab, color difference (Δ*E*), color yield [*K*/*S*], and reflectance [*R*]. The color change was evaluated using a D65 illuminant [standard] standard observer in the visible spectrum 400–700 nm. *K*/*S* represents the color yield and is evaluated using Kubelka–Munk theory.^50^

$$K/S=\left[{\left(1-R\right)}^{2}\right]/2R$$

where *K* is the absorption coefficient and *S* is the scattering coefficient.

The color space can be presented using various components of CIE l*, a*, b* that give a numerical value to identify the color more precisely (Commission Internationale d’Eclairage, CIE). The International Commission on Illumination [CIE] is the international organization on color, illumination, and color spaces, based in Vienna, Austria.^51^ The color change (Δ*E*) depends on the *l*, *a*, and *b* values, where l* indicates the lightness of the sample; the higher the value, the lighter the shade, which has a ratio scale from 0 to 100, where 0 indicates black and 100 indicates the material is white [see color space, Figure 1]. The CIE color space a* component indicates the position between red and green; positive values indicate red color, while negative values indicate green. The CIE color space b* indicates the position between yellow and blue (positive values indicate yellow, and negative values indicate blue).

### Fabric Performance

Denim fabrics were also evaluated for their durability, including the tensile strength grab method,^52^ which measures the breaking strength (N) and elongation (mm) of finished fabrics in warp and weft directions. The method applies to woven fabrics, which measures the maximum force required to rupture the fabric under specified conditions (uses Testometric equipment, which works on a constant rate of extension with one clamp being stationary and another moving at a steady speed).^52^ All the fabrics were conditioned in standard laboratory conditions (65% relative humidity and temperature 20 ± 5 °C) for 24 h before testing.^53^ As the chosen fabric was proposed for a denim garment application, the drape coefficient was determined, which measures the ability of the fabric to fall or hang over a three-dimensional form freely.^54^

### Antibacterial Tests

The antibacterial properties of finished denim fabrics were evaluated using quantitative^55^ methods with *S. aureus* strain no. ATCC 6538 (Gram-positive bacteria) and *E. coli* strain no. ATCC 10799 (Gram-negative bacteria). A 1 mL portion of test inoculum (*S. aureus* and *E. coli*) was added to the fabric swatch of diameter 4.8 cm. The bacterial strains were cultured in nutrient agar, and the percentage reduction of bacteria was determined using the formula below. The finished denim fabrics were laundered ten times using a standard ISO procedure [wash temperature of 60 °C, standard detergent (IEC), and material-to-liquid ratio of 1:50]. The antibacterial tests were repeated after ten washes.

R=[(B−A)/B]×100%

where *A* is the number of bacteria recovered from the inoculated specimen after 24 h and *B* is the number of bacteria recovered immediately after inoculation at 0 h. Statistical significance between bacterial recovery and viability pre- and postwashing was determined by one-way ANOVA with a Tukey post hoc analysis (GraphPad Prism 9.0.2). The standard error of the mean was reported for the two biological replicates.

### Test for In Vitro Cytotoxicity

An in vitro cytotoxicity test was performed using a modified BS EN ISO 10993-5:2009 ^56^ standard protocol to assess the biological safety of finished fabrics against mammalian cell lines. Briefly, human epidermal keratinocyte (HaCaT) cells were cultured in Dulbecco’s modified Eagle’s medium (DMEM) (Corning Incorporated) supplemented with 10% fetal bovine serum (FBS) (Thermo Fischer Scientific) and 2% penicillin–streptomycin (Thermo Fischer Scientific) and incubated at 37 °C in a 5% CO_2_ humidified atmosphere. Cells were grown to 80% confluency and seeded into Nunclon-delta-treated 96-well plates at 1 × 10^4^ cells per well. Extracts from finished and unfinished denim fabrics were made following ISO 10993-12, with leachate being directly made into DMEM media, and cells were exposed to a 100% extract for 24 h. CCK-8 reagent containing WST-8 (2-(2-methoxy-4-nitrophenyl)-3-(4-nitrophenyl)-5-(2,4-disulfophenyl)-2*H*-tetrazolium, monosodium salt) (Merck) was added to wells, and cells were further incubated for 4 h at 37 °C. Cell metabolic activity, as demonstrated by the bioreduction of WST-8 to formazan (orange), was detected at an absorbance of 450 nm to determine cell viability when compared to untreated control cells. The percentage cell viability was determined using the equation below, where OD_450e_ was the mean value of the measured optical density of the test extract and OD_450b_ was the mean value of the untreated control cells. Cell viability of >70% indicated minimal/acceptable cytotoxicity of the denim fabric. Statistical significance was determined using a one-way ANOVA with Tukey post hoc analysis (GraphPad Prism 9.0.2).

$$Viability\;\left(\%\right)=\frac{\left[100\times {OD}_{570e}\right]}{{OD}_{570b}}$$

### X-ray Diffraction Analysis

X-ray diffraction (XRD) measurements were performed on the denim fabric samples to obtain the structural information using a PANalytical X’Pert powder X-ray diffractometer with Cu (λ = 1.54 Å) as the source, with 45 kV voltage and 40 mA current settings. The data were collected in a continuous mode over the 2θ scan range of 10–100°, with a step size of 0.01° for 98 s per step at room temperature under ambient conditions. The samples were spun at 16 rpm during the measurements for uniform data collection. The PreFIX module on the incident beam side with the automatic divergence and fixed antiscatter slit of 4°, along with the PreFIX module on the diffracted side with the PIXcel 1D detector in scanning line mode with the programmable antiscatter slit, was used to collect the diffraction patterns from a constant irradiated length of 0.5 mm. Origin software (ver. 8.5) was used for XRD peak analysis to determine crystallite size. Scherrer’s equation^57^ was used to calculate the crystallite size:

D=Kλ/(βcos⁡θ)

where *D* is the crystallite size (λ, usually nm); *K* is the shape factor (≈0.89–1.0, commonly 0.9); λ is the X-ray wavelength (nm), for instance, Cu Kα = 0.15406 nm; β is the peak breadth (FWHM) in radians, corrected for instrumental broadening; and θ is the Bragg angle, 2θ in radians.

## Results and Discussion

### Particle Size Analysis

The particle size was monitored soon after preparation and for over 2 weeks [see Figure 2]. The particle size for the nanoemulsion Shankapushpi decreased from 107 nm “immediately after preparation” to 92 nm after the “first week” and to 38 nm after “2 weeks”. The particle size decreased consistently over time due to the presence of antioxidants in the form of free radicals in the nanoemulsion, which are unstable. This enables the reduction of the particle size of the nanoemulsion. In addition, the addition of surfactant to the nanoemulsion also reduces the particle size, assists in the ease of penetration into the fabric, and offers shelf life.^43^ The above trend of particle size was applicable to the nanoemulsion Karanja, where the particle size decreased from 106 to 93.6 nm after 1 week, and then a further reduction was observed (36.3 nm) after 2 weeks. *Millettia pinnata* oil (Karanja) is a mix of several triglycerides, and the average molecular weight of *Millettia pinnata* oil typically ranges from approximately 870 to 885 g/mol.^58^ Similarly, the molecular weight of *Evolvulus alsinoides* Linn. has been reported earlier, which is composed of various bioactive compounds such as *n*-hexadecanoic acid, cytidine, and so forth. The molecular weight can vary between 130 and 450 g/mol.^59^

**Figure 2:**
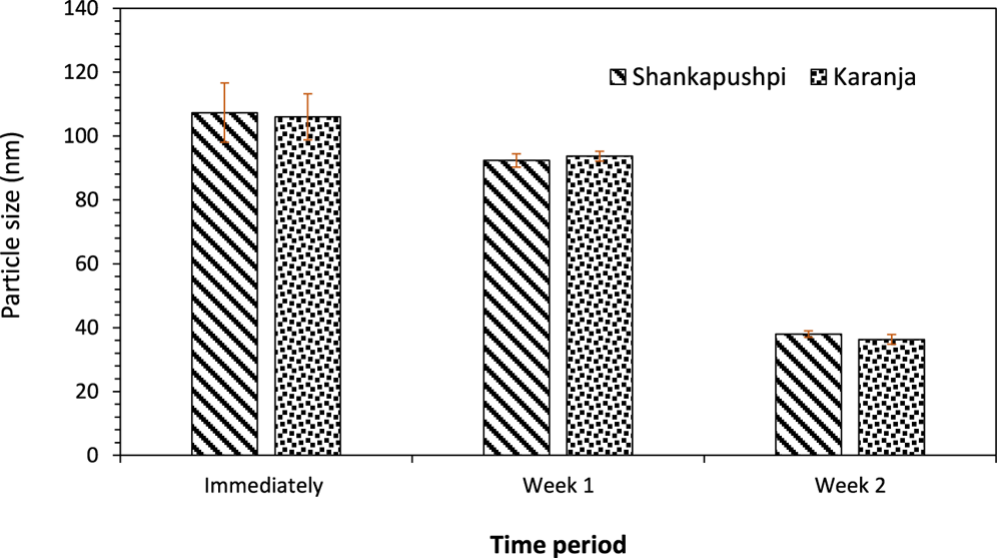
Particle size analysis.

### Thermal Stability and pH

Nanoemulsions were studied for thermal stability by placing them in a water bath maintained at 95 °C. Karanja nanoemulsions were stable at 56 ± 1 °C, while the Shankapushpi nanoemulsion was stable at 57.66 ± 1.52. Beyond this temperature, both nanoemulsions were unstable, turbid, and broke down. This trend is similar to the thermal stability of nanoemulsions studied in previous research.^43,44^ As the nanoemulsions were mixed with the surfactant–polysorbate (1:1 ratio), their surface tension was reduced, increasing the shelf life and stability. Nanoemulsions are generally unstable, and two immiscible liquids (water and oil) will break at higher temperatures. pH optimization of nanoemulsions was carried out for a ratio of 1:1 by adding 0.1% hydrochloric acid and 0.1% sodium hydroxide. pH for Karanja was 6.07 ± 0.41; Shankapushpi: 6.17 ± 0.25. pH below 7 indicates that the nanoemulsions are acidic and have a pH similar to that of common foods such as melon and honeydew.^60–62^

### The Percentage Add-On of Nanoemulsions

The percentage add-on was evaluated for both nanoemulsions. The percentage add-on for Shankapushpi (13.71%) was higher compared to that for Karanja (6.7%), Figure 3. This could be attributed to the nanoparticle size, where the Shankapushpi nanoemulsion was marginally higher than the Karanja. Scanning electron microscopy (at 3 K magnification; see Figure 4) reveals that a thin layer of nanoemulsion was applied to the fiber surface as a coating. Tiny nanoparticles are visible for Shankapushpi, whereas for Karanja, a thin layer of nanoemulsion is coated on the fabric (see Figure 4). These images also show that the percentage add-on to the fabric was a simple mechanical adsorption between the voids of the fabric structure.

**Figure 3:**
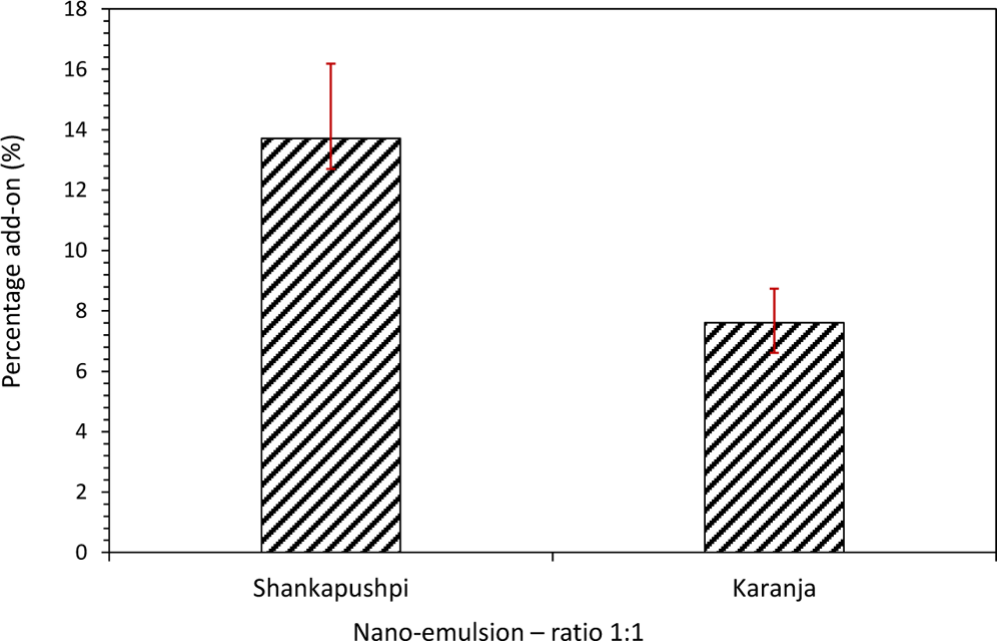
Percentage add-on for finished denim fabrics.

**Figure 4:**
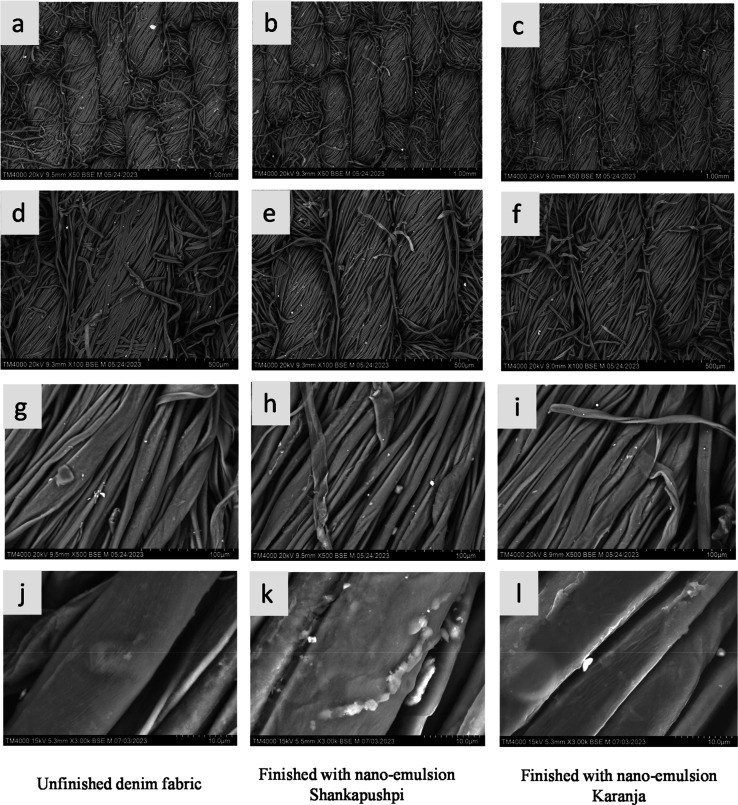
Scanning electron micrographs of unfinished denim fabric and fabric finished with Shankapushpi and Karanja. (a) Unfinished denim showing the denim fabric structure, ×50 magnification; (b) denim fabric finished with Shankapushpi nanoemulsion, ×50; (c) denim fabric finished with Karanja nanoemulsion, ×50; (d) unfinished denim, ×100 magnification; (e) denim fabric finished with Shankapushpi nanoemulsion, ×100 magnification; (f) denim fabric finished with Karanja nanoemulsion, ×100; (g) unfinished denim fabric, ×500; (h) denim fabric finished with Shankapushpi nanoemulsion, ×500; (i) denim fabric finished with Karanja nanoemulsion, ×500; (j) unfinished denim fabric, ×3000 magnification; (k) denim fabric finished with Shankapushpi nanoemulsion, ×3000; (l) denim fabric finished with Karanja nanoemulsion, ×3000.

The denim fabric is a relatively densely woven fabric with a twill weave structure. The cover factor (area covered by a set of threads) of the woven fabric was −27 (warp) and 20 (weft), indicating the fabric is a relatively open woven structure and makes it easy to penetrate the yarns and fibers within the structure. The percentage add-on is based on the mechanical interaction between the nanoemulsion and fabric structure, with no cross-linking. It depends on the particle size of the nanoemulsion. This was similar to previous research^34^ on lightweight cotton fabrics, which also had a higher percentage add-on on a 20-gsm woven fabric than on a 60-gsm fabric. This was due to the cover factor (the area of fabric covered by a set of threads) of 20 gsm being less (the cover factor was 21 in the warp direction) than the 60 gsm fabric (the cover factor was 63 in the warp direction). In the denim fabric, the cover factor is composed of warp and weft directions. This revealed that the percentage add-on for the denim fabric with an open structure and finishing was related to the mechanical adsorption of nanoemulsions in the interstices of the fibers in the fabric.

### Physical Properties of the Fabric and Surface Morphology

Denim fabrics were finished with nanoemulsion 1 (*Evolvulus alsinoides* (Shankapushpi), curry leaves, and coconut oil) and nanoemulsion 2 (*Millettia pinnata* (Karanja), curry leaves, and coconut oil). The denim fabric used in the study was a 3/1 warp-faced twill woven fabric in dark indigo blue. It is an indigo-dyed fabric ideal for outerwear, such as jeans, with a fabric weight of 410 gsm and a thickness of 0.73 mm. The surface morphology of the fabric was analyzed using SEM [scanning electron microscopy]/EDX [energy-dispersive X-ray diffraction analysis]. Surface morphology analysis revealed that the cotton fibers (warp yarns) of unfinished fabrics were round and ribbon-shaped, and the fibers were evenly twisted in an anticlockwise direction (“Z” twist) [see image “g” from SEM micrographs]. However, the SEM micrographs showed a marginal flattening of fibers due to finishing denim fabrics with nanoemulsions [see images “h” and “i” from SEM micrographs, Figure 4]. This is mainly due to the stretching of fabrics between nip rollers during the finishing of fabrics in a continuous method (pad-dry-cure) with nanoemulsions. EDX analysis was used to determine the elemental composition, revealing minor traces of sodium, aluminum, and silica in both nanoemulsions. This is mainly due to nip rollers that are coated with silicone rubber and wet-pickup rollers made of aluminum (Figure 5).

**Figure 5:**
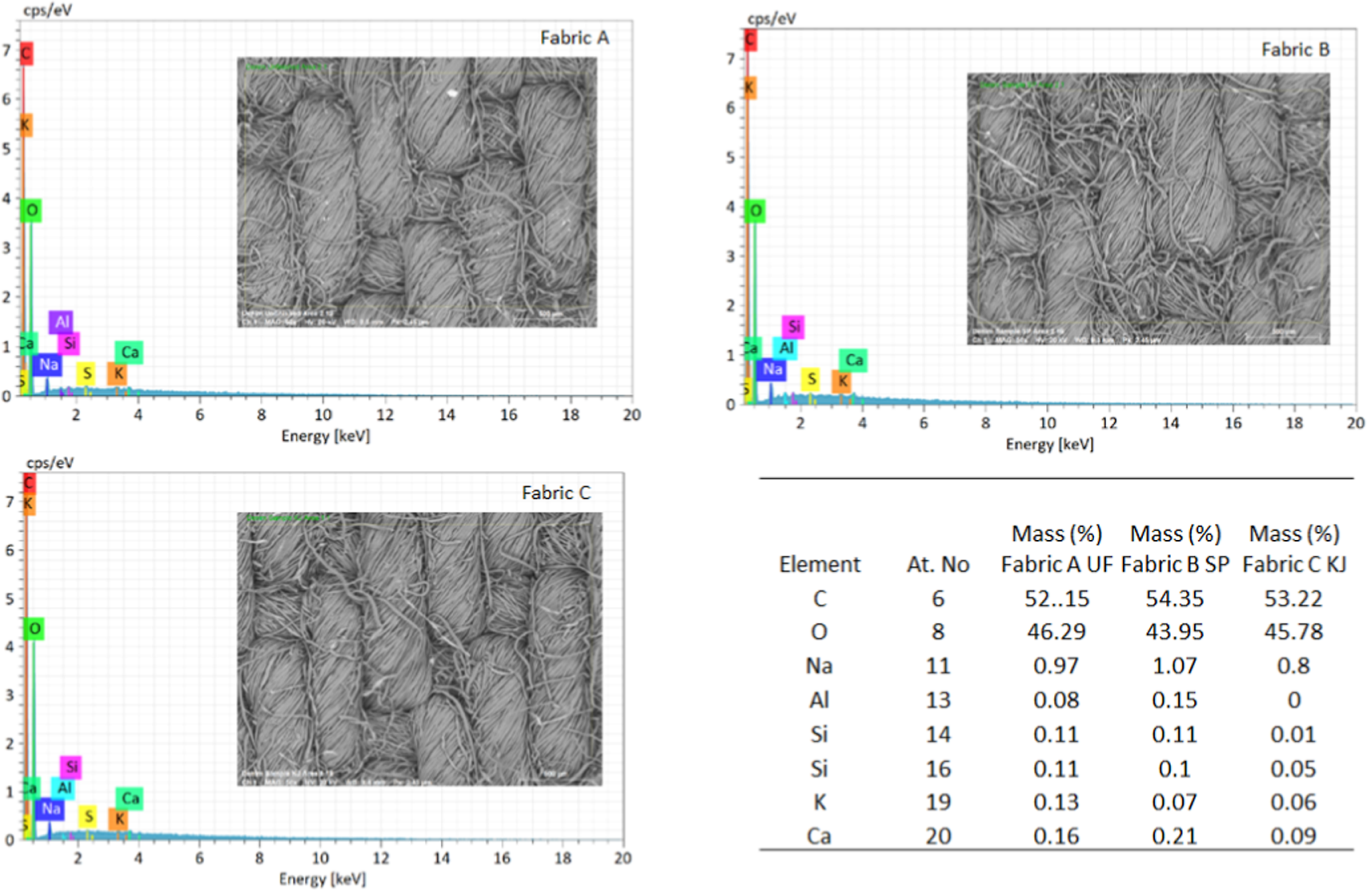
Energy-dispersive X-ray spectroscopic analysis [EDX]—(A) unfinished fabric and (B) finished with Shankapushpi and (C) Karanja.

### ATR-FT-IR

Herbal oils are concentrated solutions of volatile compounds consisting of complex, homogeneous mixtures of various compounds, with more than 100 constituents present in each species (taxon). Their FT-IR spectra are complex due to the spectra of individual components overlapping and the mixing of various vibrational modes. Although contributions of major components never exceed 25% of the total content, those compounds in essential oils that occur at low concentrations (<1) do not influence the ATR-IR spectrum significantly. Thus, ATR-FT-IR spectra of the essential oil samples exhibit characteristic spectral fingerprints that can be used to discriminate between different plant species and chemotypes.

ATR-FT-IR absorption spectra of the concentrated essential oils show the expected characteristic C–H stretch (∼2922 cm^–1^), C=O stretch (∼1743 cm^–1^), broad O–H stretch (∼3006 cm^–1^ and 3008 cm^–1^), and C–O stretch (∼1162 cm^–1^) of terpenoid components present in the essential oils (Figure 6). It is interesting to note that in the case of both the nanoemulsions, peaks were not observed between 590 and 1462 cm^–1^ (Figure 7). In the case of Karanja and Shankapushpi oils, peaks were observed between 590 and 3006 cm^–1^, which are common in vegetable oils. However, the peak at 1743 cm^–1^ represents the existence of an ester and aldehyde. At 3336 cm^–1^ for both nanoemulsions, it is associated with the hydroxyl or alcohol group, and this could have occurred during the emulsion formation process. The rest of the peaks that are C=O ester and aldehyde groups observed at 1743 cm^–1^ are strong and are not affected during the emulsion formulation process. However, the triple-bonded carbon C≡C at 2105 cm^–1^ is a strong bond and could be associated with antimicrobial efficacy. It is also noted in both the nanoemulsions, Karanja and Shankapushpi. Carbon–carbon peaks are variable in position because they are associated with hydroxyl groups. The spectral assignments for both oils and nanoemulsions are presented in Table 2.

**Figure 6:**
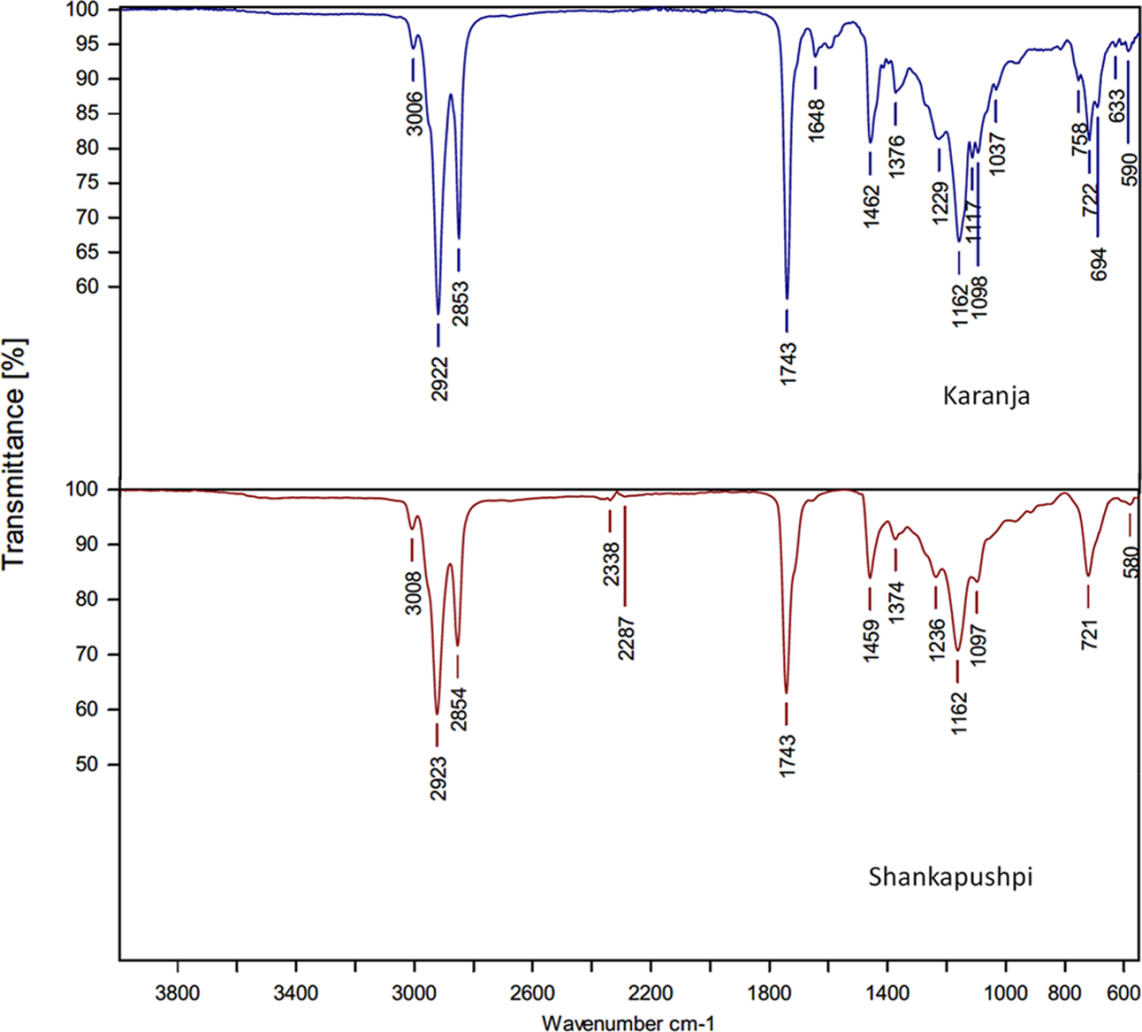
ATR-FT-IR spectra of Karanja and Shankapushpi.

**Figure 7:**
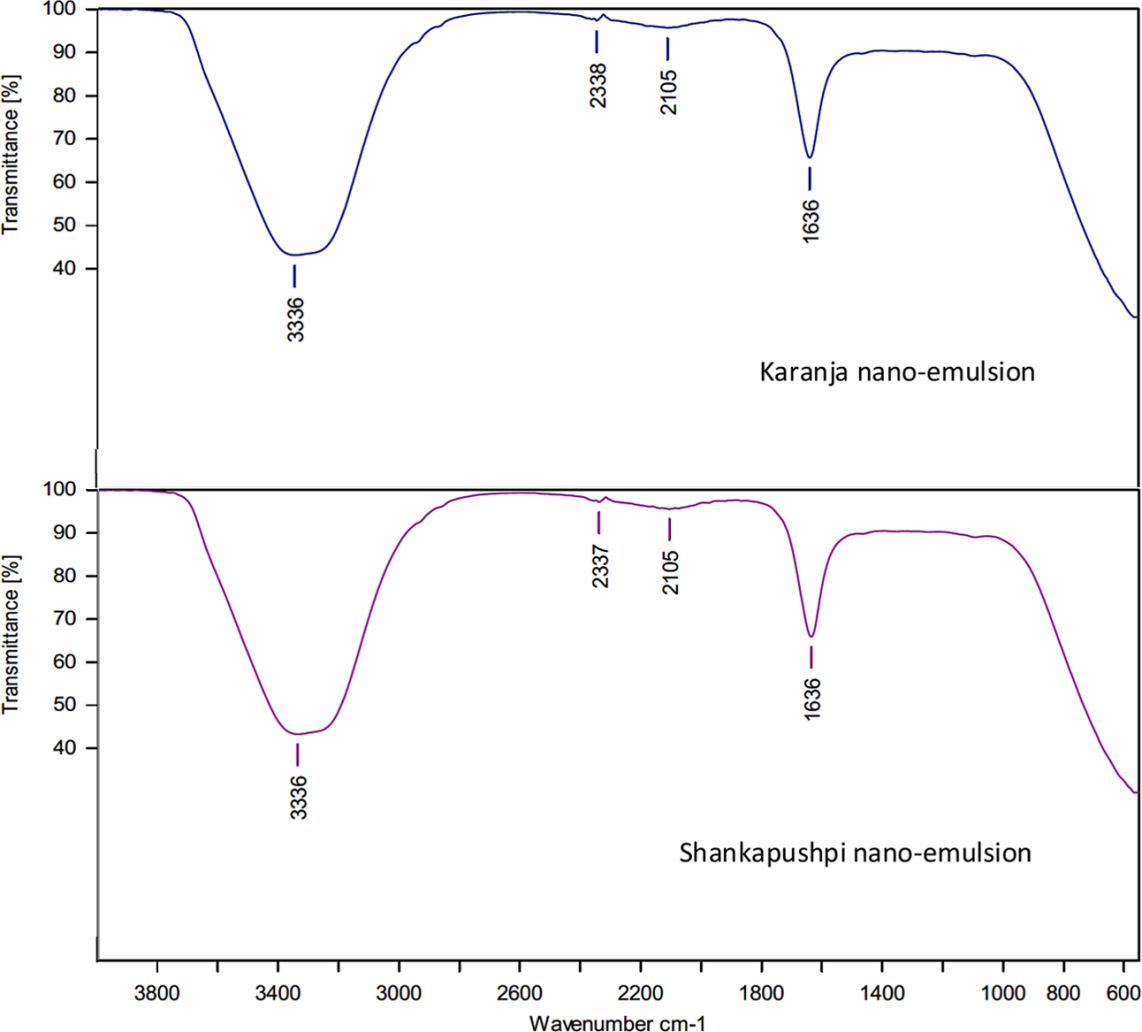
ATR-FT-IR spectra of Karanja and Shankapushpi nanoemulsions.

**Table 2:** Indexing of FT-IR Absorption Peaks (in cm^–1^)

peak position [cm^–1^]			peak position [cm^–1^]		
Karanja	Karanja nanoemulsion	assignment	Shankapushpi	Shankapushpi nanoemulsion	assignment
590		out-of-plane deformation of C–H	580		out-of-plane deformation of C–H
633		C–C–C			
694		mono and polyclinic substituted aromatics group (O–H group)			
722		=C–H bending	721		=C–H bending
758		mono and polyclinic substituted aromatics group (O–H group)			
1037		C=O stretching and deformation			
1098		C=O stretching and deformation	1097		C=O stretching and deformation
1117		C=O stretching and deformation			
1162		C–O stretching mode of the C–OH group of esters	1162		C–O stretching mode of the C–OH group of esters
1229		C–O stretching mode of the C–OH group of esters	1236		C–O stretching mode of the C–OH group of esters
1376		O–CH_2_ (glyceride group)	1374		O–CH_2_ (glyceride group)
1462		alkyne (Ca·C deformation)	1459		alkyne (Ca·C deformation)
1648	1636	unsaturated aldehyde and ketones		1636	unsaturated aldehyde and ketones
1743		C=O (existence of ester)	1743		C=O (existence of ester)
	2105	C≡C stretch		2105	C≡C stretch
			2287		C=N=O asymmetric stretch vibration
	2338		2338	2338	CO_2_
2853		CH_3_ and CH_2_ asymmetric stretching (methyl group or methylene group)	2854		CH_3_ and CH_2_ asymmetric stretching (methyl group or methylene group)
2922		C–H stretching or CH_3_ and CH_2_ asymmetric stretching (methyl group or methylene group)	2923		C–H stretching or CH_3_ and CH_2_ asymmetric stretching (methyl group or methylene group)
3006		CH_3_ and CH_2_ asymmetric stretching (methyl group or methylene group) or high degree of unsaturation(i.e., fatty acids with a greater number of *cis*-alkene −HC=CH– bonds)	3008		CH_3_ and CH_2_ asymmetric stretching (methyl group or methylene group) high degree of unsaturation(i.e., fatty acids with a greater number of *cis*-alkene – HC=CH– bonds)
	3336	alcohol group (maybe associated with the –OH group during nanoemulsion)		3336	alcohol group (maybe associated with the –OH group during nanoemulsion)

During the nanoemulsion process, a thin film is formed on the surface that does not have a C–H bending, and carbon only contains unsaturated aldehyde and ketones. At 2922 cm^–1^, C–H stretching or CH_3_ and CH_2_ asymmetric stretching (methyl or methylene groups) can be observed. At 1376 cm^–1^, it is associated with a glyceride group. It is also worth mentioning that in the proximate analysis, Karanja contains 72.25% volatile compounds. The ultimate analysis indicates that Karanja contains carbon [51%], hydrogen [3%], and nitrogen [6%]. Since the primary component is carbon, this supports the antimicrobial properties of Karanja.^63^

Earlier reports on Karanja seed oil also reported FT-IR peaks relating to the alcohol group at 3212 cm^–1^, aldehydes at 1711 cm^–1^, and alkynes at 1464 cm^–1^.^64^ Similarly, *Convolvulus pluricaulis* (Shankapushpi) was reported for its functional groups—alcohol, carboxylic acids, alkynes, and aldehydes, much like the present study.^63^ Shankapushpi has fewer peaks than Karanja and fewer OH groups and can be more effective with antimicrobial properties. The peak at 2287 cm^–1^ is observed only in Shankapushpi and corresponds to the C=N=O asymmetric stretch; this type of bonding is common in most antibiotics. The literature indicates that drying the sample has no or a minimal effect on the peak position or the FT-IR wavelength. The peak position of organic components remains the same whether it is freeze-dried, air-dried, or vacuum-dried. For instance, in the case of aloe gel, which contains many organic components and minerals, the drying process leads to partial disruption of organic molecules and degradation of polysaccharides.^65^ There is no effect on the wavelength.

### Antibacterial Assays

The antibacterial assays revealed a percentage reduction in microorganisms (both Gram-positive and Gram-negative bacteria) following exposure to mechanically finished nanoemulsion denim, both before washing and after 10 washes following 24 h of bacterial exposure to each material. There was a 99.73% (for *S. aureus*) and 99.74% (for *E. coli*) decrease in the growth of microorganisms after exposure to Karanja nanoemulsion before washing (Table 3), which was a significant reduction in bacterial viability (*p* < 0.0001) as determined by a one-way ANOVA with a Tukey’s post hoc analysis. After ten washes, there was a 0.64% and 0.65% reduction in antimicrobial activity compared to the observed activity of unwashed denim against *S. aureus* and *E. coli*, respectively, which was anticipated and likely due to the removal of some surface finishing of nanoemulsions following the mechanical agitation during the washing process. However, the minor loss of activity was not statistically significant compared to the antimicrobial activity of unwashed denim (*p* > 0.05). After exposure of bacteria to Shankapushpi-finished denim, there was a statistically significant 99.77% and 99.73% reduction (*p* < 0.0001) in bacterial viability for *S. aureus* and *E. coli*, respectively, after 24 h of incubation. These antimicrobial activity profiles were similar to those observed for the Karanja-finished fabrics with no statistical difference in activity between the two types of finishes (*p* > 0.05). After ten washes of Shankapushpi-finished denim fabric, there was a slight decrease in antibacterial activity of 0.38% and 0.52% against *S. aureus* and *E. coli*, respectively, but this was not statistically significant compared to the antibacterial activity of the corresponding unwashed denim (*p* > 0.05). Overall, both Karanja- and Shankapushpi-finished denim demonstrated significant antimicrobial activity despite ten washes, which demonstrated the durability of the mechanical finishes. For bacteria exposed to denim with digitally printed finishings, there was no observable decrease in bacterial viability, but likewise, there was also no statistically significant increase in bacterial growth over a 24 h period (*p* > 0.05). Based on the above findings, further work will address the detailed durability and antibacterial efficacy of the garments after repeated washing with both digital and mechanical finishes.

**Table 3:** Antibacterial Assays Demonstrating a Reduction of Microorganisms (%) before and after Washing Mechanically Finished Denim Samples

s. no	fabric finishing	condition	test culture	reduction of micro-organisms (%) *R* (standard error of mean)
1	denim fabric finished with Karanja nanoemulsion	before wash	*S. aureus*	99.73 (±0.01)
			*E. coli*	99.74 (±0)
		after 10 washes	*S. aureus*	99.09 (±0.02)
			*E. coli*	99.09 (±0.01)
2	denim fabric finished with Shankapushpi nanoemulsion	before wash	*S. aureus*	99.77 (±0.02)
			*E. coli*	99.73 (±0.01)
		after 10 washes	*S. aureus*	99.39 (±0.01)
			*E. coli*	99.21 (±0.01)

It can be noted that GC–MS [gas chromatography and mass spectroscopy] analysis of Karanja extract showed the presence of saturated fatty acids [such as caprylic acid, lauric acid, myristic acid, and 1-monolaurin] that prohibit the growth of bacteria.^44^ In addition, the GC–MS analysis of Shankapushpi revealed the presence of various compounds such as 4-(3-hydroxybutyl) phenol, hexadecanoic acid, 9,12-octadecadienoic acid, and ethyl oleate.^47^ Ethyl oleate is formed by the condensation of the carboxyl group of oleic acid with the hydroxy group of ethanol and is a long-chain fatty acid ethyl ester.^66^ Oleic acid possesses antibacterial activity against various Gram-positive bacterial species.^67^

### X-ray Diffraction Analysis

X-ray diffraction (XRD) study of denim samples revealed unique peaks for cellulose, coumarin, and bathophenanthroline. Peaks at 14.77°, 16.70°, and 22.67° confirmed the crystalline structure of cellulose (JCPDS no. 050-2241, Joint Committee on Powder Diffraction Standards). Additional peaks at 33.49°, 47.39°, and 48.14° confirmed the successful inclusion of coumarin (JCPDS no. 048-2297) in the cellulose matrix. Bathophenanthroline (JCPDS no. 017-1190) was detected with a peak at 29.27° in the Shankapushpi digital print, Shankapushpi mechanical finish, and unfinished denim samples, indicating that it forms a complex structure within the denim fabric. It was interesting to observe from Figure 8 that this peak was suppressed in the Karanja-finished denim sample. This compound may have been noticed following the dyeing and processing of denim fabrics. Coumarin is expected to enhance denim performance by adding fluorescent, UV-protective, and antibacterial properties.^68–70^ Furthermore, bathophenanthroline may aid in metal-ion complexation (the ability to bind other metal compounds) and colorimetric sensing (the ability to detect different colors), enabling the development of smart textiles.^71,72^ In summary, the presence of cellulose, coumarin, and bathophenanthroline indicates successful functional modification of the denim fabric. It suggests other potential uses, including UV-protective finishing and sensor-enabled textiles.

**Figure 8:**
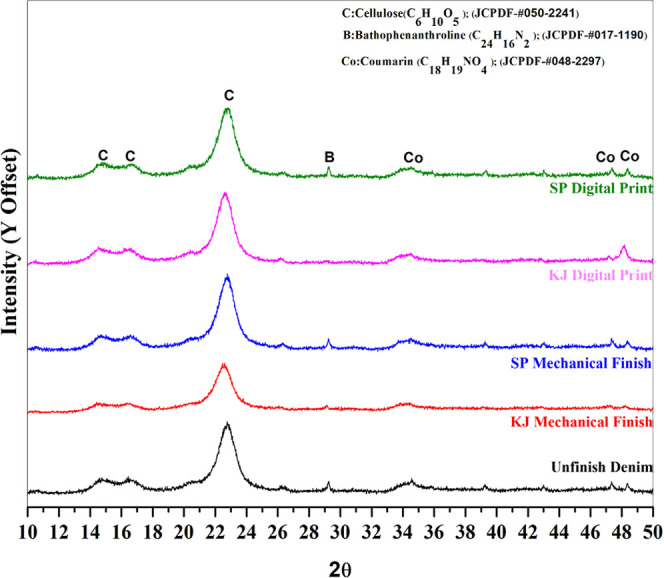
X-ray diffraction spectra of various denim fabrics.

The Scherrer equation was used to estimate the crystallite size of denim fabric samples by using X-ray diffraction (XRD) and Cu Kα_1_ radiation (λ = 1.540598 Å). The diffraction peaks at 22.5° to 22.7° showed significant widening, indicating the presence of nanocrystalline structures. The computed crystallite sizes varied among samples, ranging from 4.80 to 5.61 nm (Table 4). The Karanja digital print sample had the highest crystallite size (5.61 nm), whereas the Shankapushpi digital print sample had the smallest size (4.80 nm). The average crystallite size across all samples was approximately 5.05 nm, indicating the presence of tiny nanocrystals within the denim material. This nanoscale crystallite size indicates that various finishing and printing processes may alter the surface morphology of the fabric and structural compactness.

**Table 4:** XRD Analysis and Crystallite Measurements

s. no.	sample	2θ (angle)	full-width half-maximum (fwhm)	crystallite size (nm)
1	unfinished denim	22.72	1.662	4.87
2	Karanja digital print	22.59	1.444	5.61
3	Shankapushpi digital print	22.70	1.687	4.8
4	Karanja mechanical finish	22.50	1.647	4.92
5	Shankapushpi mechanical finish	22.70	1.612	5.03

### Tensile Strength

Denim fabrics finished with the nanoemulsions were evaluated for their durability, tensile strength, and breaking elongation in warp and weft directions. It could be noted that for denim fabrics finished with Karanja, the fabric had a decrease in strength in the warp direction when compared to the tensile strength before finishing. In the weft direction, there was a marginal increase in the strength when compared with the unfinished denim fabrics. In the case of the fabric finished with Shankapushpi, there was a marginal increase in the strength in warp and weft directions [Table 5]. It could be noted that the finishing of fabrics with the nanoemulsion is due to the simple mechanical adsorption of the fabric structure, whereby the nanoparticles remain in the voids of the fabric structure. The pH of the nanoemulsions [Karanja 6.07 ± 0.41 and Shankapushpi 6.17 ± 0.25] is acidic in nature, and the cotton fibers are sensitive to acids and lose their strength. This trend is similar to previous research, in which organic cotton fabrics finished with nanoemulsions had a higher tensile strength than unfinished fabrics.^44^ The breaking extension is the maximum tensile force recorded when the specimen breaks, and it depends on the cross-sectional area of the material.^73^ The breaking extension of the finished and unfinished fabric was unchanged in warp directions, while in weft directions, there was a minor increase in the breaking extension.

**Table 5:** Tensile Strength and Breaking Extension

		tensile strength (N)		elongation (mm)	
type	denim fabric	warp	weft	warp	weft
mechanical finishing	unfinished fabric	546.22 (±5.36)	398.61 (±47.66)	40.66 (±2.45)	24.50 (±1.66)
	finished with Karanja nanoemulsion	534.78 (±27.49)	510.08 (±46.26)	40.99 (±0.41)	30.45 (±4.44)
	finished with Shankapushpi nanoemulsion	547.33 (±0.68)	538.66 (±15.81)	40.51 (±1.87)	29.20 (±1.77)
digital printing	finished with Karanja nanoemulsion	410.05 (±26.35)	230.04 (±57.36)	37.77 (±5.81)	18.88 (±0.39)
	finished with Shankapushpi nanoemulsion	423.30 (±4.85)	395.83 (±3.46)	42.16 (±0.45)	19.84 (±0.93)

### Fabric Drape and Garment Development

The finished denim fabrics were evaluated for drapeability. It is a characteristic of a material to fall or hang over a three-dimensional form freely.^74^ The drape coefficient value of 19% indicates a pliable fabric, while 61% indicates a medium fabric and 95% indicates a stiff fabric.^54^ Fabric finished with Karanja had a coefficient of 84%, while Shankapushpi was 91%, showing that denim fabric finished with Karanja is comparatively more pliable to fabric finished with Shankapushpi. In addition, it is worth mentioning that both the finished fabrics had their coefficient lower than the unfinished fabric (94%), indicating that adsorption of nanoemulsions enabled the fabric to decrease its stiffness marginally. The garment was developed into a pair of jeans (five pockets) fitting a female size 12, Figures 9 and 10 Karanja and Shankapushpi, respectively. The garment fit was evaluated on a mannequin (Alvaform—soft series). The donning and doffing of the garment were easier to handle, and it fit well on the mannequin, indicating that denim fabrics finished with nanoemulsions did not affect the fabric handle and improved the fabric drape marginally.

**Figure 9:**
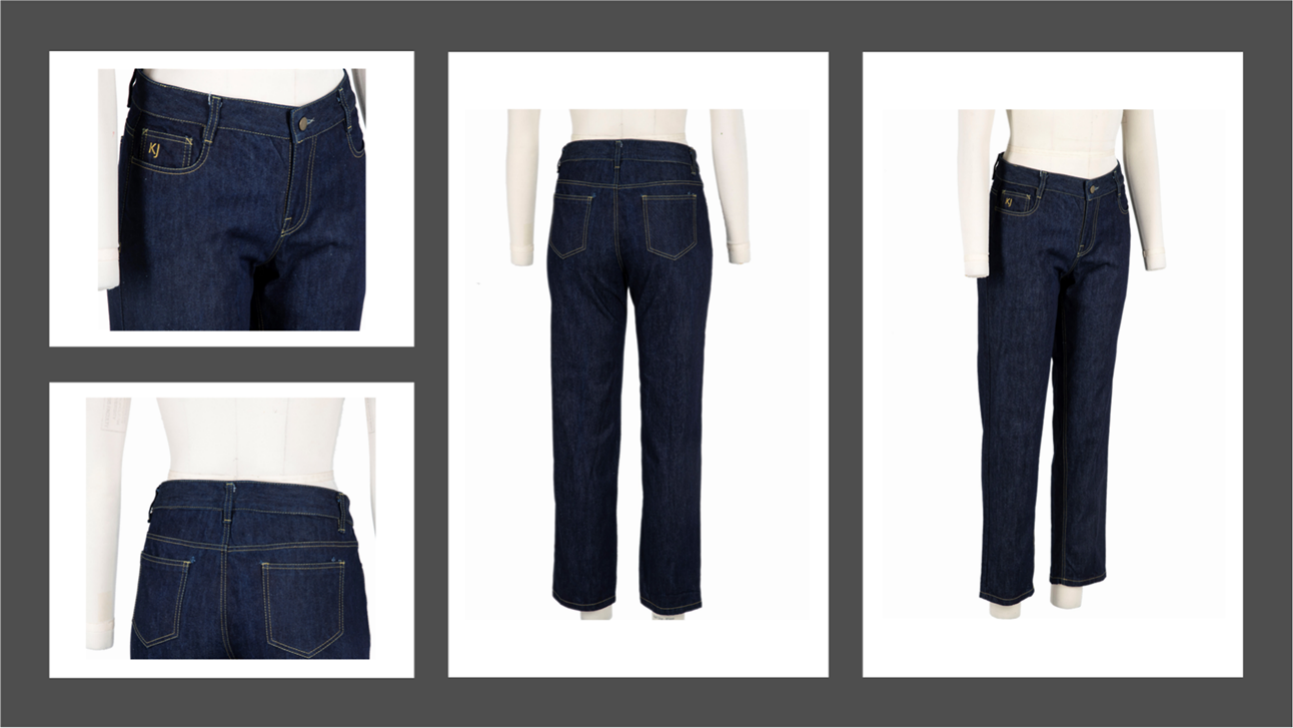
Denim garment developed and finished with Karanja nanoemulsion (author’s own image: courtesy of Richard Kelly).

**Figure 10:**
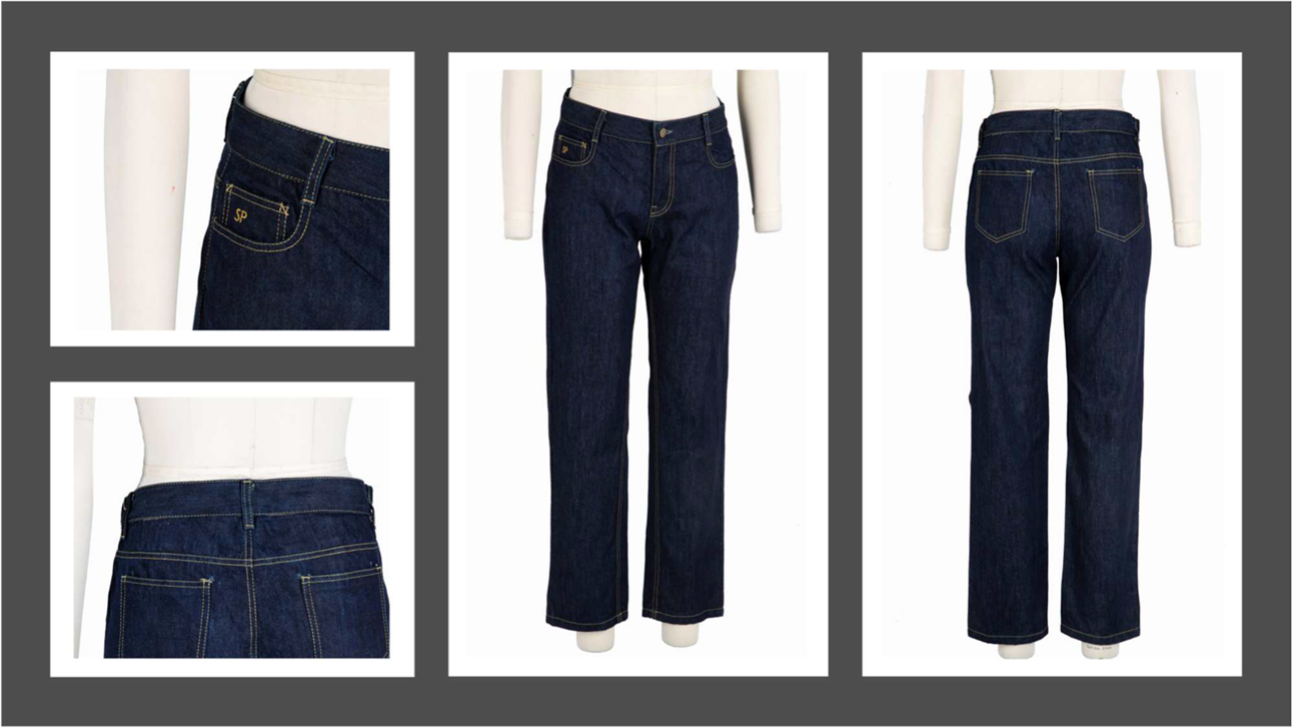
Denim garment developed and finished with Shankapushpi nanoemulsion (author’s own image: courtesy of Richard Kelly).

### In Vitro Cytotoxicity of Denim Fabrics

ISO 10993-5 ^56^ is an internationally recognized standard test method that provides guidelines for conducting in vitro cytotoxicity assays to determine the biological safety of materials. It is essential to conduct in vitro cytotoxicity assays on denim fabric due to the near-skin application of the fabric material. The indirect cytotoxicity test aimed to assess the possible harmful effects of substances released from the finished fabrics. Extracts from unfinished and denim fabrics finished with different herbal nanoemulsions were exposed to mammalian human epidermal keratinocytes (HaCaT) as described in a modified version of ISO 10993-5. The percentage viability of cells exposed to the unfinished fabric extracts was 103.58% ± 0.008% at 100% extract elution (Figure 11A), indicating that cell metabolic activity was slightly increased in the presence of the extract compared to that of untreated control cells. Likewise, the cell viability for denim fabric extracts mechanically finished with Shankapushpi and Karanja nanoemulsions was 97.66% ± 0.01% and 105.29% ± 0.004%, respectively (Figure 11B,D). There was no significant difference in viability (*p* > 0.05) when these extracts were compared to unfinished denim (Figure 11A) as determined by a one-way ANOVA with Tukey’s post hoc analysis. Cell viability following exposure to extracts from denim finished with digitally printed Shankapushpi and Karanja nanoemulsions was recorded as 111.98% ± 21.94% and 117.91% ± 15.04%, which was noticeably higher than that of the unfinished and mechanically finished denim. However, this increase in cell viability was not statistically significant (*p* > 0.05).

**Figure 11:**
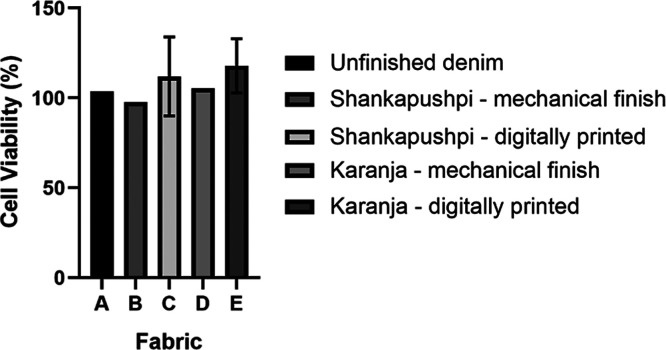
In vitro mammalian cytotoxicity assay of (A) unfinished denim, (B) denim with Shankapushpi nanoemulsion mechanical finish, (C) Shankapushpi nanoemulsion with digitally printed finish, (D) Karanja nanoemulsion mechanical finish, and (E) Karanja nanoemulsion with digitally printed finish. Cell viability (%) was determined from *n* = 3 biological replicates, with error bars (not visible in some cases due to low values) representing the standard error of the mean.

Given the observed high cell viability at 100% fabric extract elution, no further dilution assays were required, as it was predicted that they would yield similar or higher cell viability. Viability of >70% is considered an acceptable level of cytotoxicity as defined by ISO 10993-5; therefore, these finishes are biocompatible and highly suited for near-skin fabric applications.

### Color Analysis

A positive “*L*” value (21.73) for unfinished denim indicates that the fabric is darker in shade; a positive “*a*” value (1.0) suggests that the fabric has a red tint, a negative *b* value (−6.5) indicates the fabric is blue, and finally a positive chroma value (6.57) suggests that the fabric is red in color; see Table 6. In the case of Shankapusphi-finished denim, the fabric has a green and red tint. A negative value of Δ*L* indicates the fabric is darker; a positive value of Δ*a* suggests the fabric has a red tint, while Δ*h*, which shows chroma, shows that it is red in color. For the remaining fabric samples, the Karanja mechanically finished, Shankapushpi, and Karanja digitally printed samples, the Δ*L*, Δ*a*, Δ*b*, and Δ*E* values were marginally different, indicating that the finished samples were darker, had a red shade, and retained a dark blue color.

**Table 6:** Color Analysis CIE *L a b* for Unfinished and Finished Denim^a^

batch name	lightness or darkness value [CIE L*, Δ*L*]	difference on the red/green axis [CIE a*, Δ*a*]	difference on the yellow/blue axis [CIE b*, Δ*b*]	difference in chroma [CIE *C* Δ*C*]	Hue [CIE *h* Δ*H*]	total color difference [CIE Δ*E*]	description
color analysis CIE L* a* b* color illuminant *D* 65 10°							
unfinished denim	21.73	1.00	–6.50	6.57			the measured sample is darker, less saturated, and more red
Shankapushpi mechanical finished	–1.27	0.90	0.06	0.13	0.89	1.55	
Karanja mechanical finished	–2.03	0.57	–1.53	1.61	0.30	2.60	
Shankapushpi digital finished	–1.54	0.70	0.09	0.05	0.71	1.70	
Karanja digital finished	–0.96	0.43	0.27	–0.18	0.47	1.08	

a where l* indicates the lightness of the sample; the higher the value, the lighter the shade, which has a ratio scale from 0 to 100, where zero means black and 100 indicates the material is white. The CIE color space a* component indicates the position between red and green; positive values indicate red color, while negative values indicate green. The CIE color space b* indicates the position between yellow and blue (positive values indicate yellow, and negative values indicate blue). The total color difference (Δ*E*) depends on *l*, *a*, and *b* values.

The reflectance values from the spectrophotometer provide the depth of the shade in the visible spectrum. For unfinished denim and the remaining finished samples, peaks were identified at 400–420 nm, indicating that the fabric is blue in color. Low peak values were observed between 580 and 620 nm, indicating that the fabric had a lower green and yellow tint. Further peaks at 700 nm indicate that the fabric is red. Much like reflectance values, it can be noticed from Figure 12 for the *K*/*S* value, across all the samples, which shows the color yield, where high peaks are noticed between 400 and 420 nm, showing the fabric is blue in color, and low peaks between 540 and 680 nm, showing the fabric has fewer green, yellow, and orange shades. The high peak at 700 nm indicates the fabric has a red color. The color difference analysis between unfinished denim and mechanical and digitally printed denim samples was marginally different, indicating the color was not affected by finishing denim fabric with nanoemulsions. Such *K*/*S* value and CIE L*, a*, and b* were also reported to show color yield on denim fabrics where natural cellulase treatment was used to improve the desired worn and aged effect.^73^ A minor color difference (Δ*E*) was anticipated after enzyme treatment of denim fabrics, as observed in this study, which used nanoemulsions.

**Figure 12:**
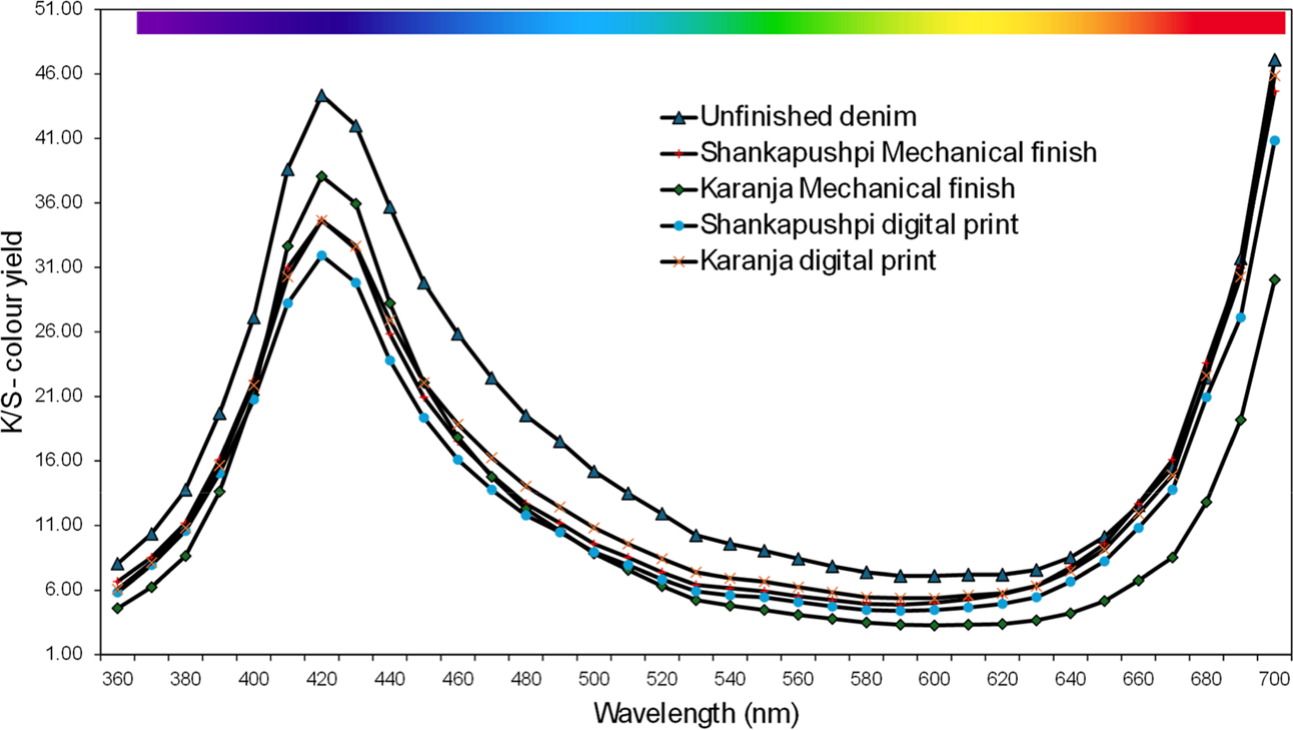
Color spectrophotometer—*K*/*S*—color yield for denim fabric samples.

## Conclusions

A heavyweight denim fabric suitable for trousers was finished using a continuous method combined with digital printing that utilized nanoemulsions derived from a blend of *Millettia pinnata* (Karanja), curry leaf, and coconut oil, as well as *Evolulus alsinoides* (Shankapushpi), curry leaf, and coconut oil. The nanoemulsions were characterized for their nanoparticle size, thermal stability, and percentage add-on. Notably, the particle size significantly decreased from the initial production over a span of 2 weeks. The presence of antioxidants in the form of free radicals within the nanoemulsion contributed to particle size reduction, while the addition of a surfactant further reduced particle size and facilitated easier penetration into the fabric interstices. The nanoemulsions developed in this research were stable, leading to nanoparticle deposition on the fiber surface without cross-linking. The percentage add-on of nanoemulsions was attributed to a mechanical interaction with the interstices of the fabric fibers. Scanning electron microscopy analysis revealed a thin layer of nanoparticles deposited on the fabric, which did not significantly alter the overall surface morphology, except for a marginal flattening of the fibers.

The attenuated total reflectance (ATR) spectra for both nanoemulsions were similar, featuring distinct C–H, C=O, and C–O stretches characteristic of essential oils. A triple-bonded carbon was detected in both the Karanja and Shankapushpi nanoemulsions, which is associated with antimicrobial properties. It can be inferred that no chemical interactions occurred after finishing denim fabrics with these nanoemulsions. Energy-dispersive X-ray (EDX) analysis of the finished fabrics revealed that they are free of harmful elements. The antibacterial efficacy of mechanically finished denim fabrics treated with Karanja and Shankapushpi was impressive, effectively preventing microbial growth, with a percentage reduction in microorganisms ranging from 99.77% to 99.09% against both Gram-positive and Gram-negative bacteria. Mechanical finishing provided durable antimicrobial protection for 10 washes, whereas digitally printed finished fabrics lacked antimicrobial activity and did not inhibit bacterial growth. Further studies are needed to determine the appropriate antimicrobial finishing concentrations for use in digital printing. Nevertheless, digital printing methods demonstrated savings in the volume of used nanoemulsions, processing time, and energy resources while maintaining overall satisfactory performance. The washing process slightly removed the surface deposition of the nanoemulsion coating from the fabric.

The tensile strength of the finished fabrics was not significantly affected by either nanoemulsion, demonstrating that the finished fabrics retained adequate strength in both the warp and weft directions. The color difference analysis, color yield (*K*/*S*), and CIE L*a*b* values between the unfinished denim and the mechanically and digitally printed denim samples were only marginally different, indicating that the finishing process with nanoemulsions did not adversely affect the fabric’s color. X-ray diffraction (XRD) analysis showed peaks confirming the crystalline structure of cellulose and the presence of phytochemical compounds, particularly coumarin, which has antibacterial effects. The average crystallite size across all samples was approximately 5.05 nm, indicating the presence of tiny nanocrystals within the denim fabric. The denim finished with the Karanja nanoemulsion had a relatively softer handle than the fabric finished with Shankapushpi and the unfinished denim. Karanja-finished denim exhibited excellent drape and remained softer than unfinished denim, which was noticeable during garment making and when the garment was donning or doffing on a mannequin. Both mechanical and digitally printed finishes on the denim fabrics were noncytotoxic and did not significantly affect mammalian cell viability, highlighting the product’s safety profile. These findings suggest that finishing denim fabrics with plant-based nanoemulsions is sustainable, environmentally friendly, safe for human skin, and free from harmful chemicals.
